# Protocol for high-precision CRISPR-Cas12a-based SNV detection on synthetic DNA, cell line cfDNA models, and liquid biopsies

**DOI:** 10.1016/j.xpro.2025.103696

**Published:** 2025-03-14

**Authors:** Kavish A.V. Kohabir, Jasper Linthorst, Rob M.F. Wolthuis, Erik A. Sistermans

**Affiliations:** 1 Department of Human Genetics, Amsterdam UMC Location Vrije Universiteit Amsterdam, Amsterdam, the Netherlands; 2 Amsterdam Reproduction & Development, Amsterdam, the Netherlands; 3 Imaging and Biomarkers, Cancer Center Amsterdam, Amsterdam, the Netherlands; 4 Amsterdam Institute for Immunology and Infectious Diseases, Amsterdam, the Netherlands; 5 Cancer Biology and Immunology, Cancer Center Amsterdam, Amsterdam, the Netherlands

**Keywords:** cell biology, cell culture, cancer, genetics, CRISPR, biotechnology and bioengineering

## Abstract

CRISPR-based diagnostics (CRISPRdx) offer promising tools for rapid and cost-effective genetic testing, but achieving single-nucleotide fidelity remains a challenge. Here, we present a protocol for high-precision detection of single-nucleotide variants (SNVs) using a Cas12a-based approach. We describe how to apply our publicly available ARTEMIS algorithm to identify targetable SNVs, design optimized CRISPR RNAs (crRNAs), and perform fluorescence-based CRISPRdx assays on synthetic DNA, cell line-derived cell-free DNA (cfDNA), and liquid biopsy samples.

For complete details on the use and execution of this protocol, please refer to Kohabir et al.^1^

## Highlights

• Use ARTEMIS to design crRNAs optimized for Cas12a-based SNV detection
• Validate crRNA specificity using synthetic DNA and fluorescence-based assays
• Assess performance on cfDNA derived from cultured cell line models
• Evaluate CRISPR-based diagnostics on liquid biopsy-derived cfDNA samples

## Abstract (editor highlights)

Publisher’s note: Undertaking any experimental protocol requires adherence to local institutional guidelines for laboratory safety and ethics.

## Graphical abstract

**Figure:**
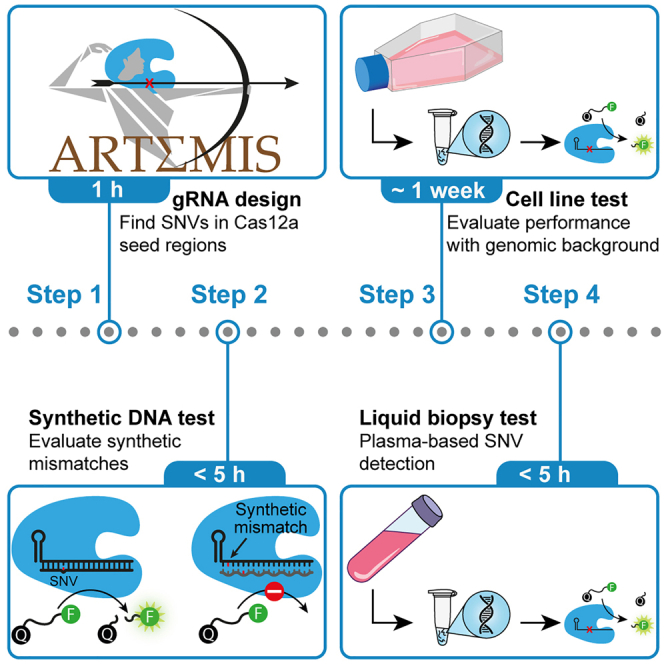

## Before you begin

Collateral activity Type V and Type VI Clustered Regularly Interspaced Short Palindromic Repeats (CRISPR)-associated (Cas) proteins present promising opportunities for genetic testing by offering cost-effective, rapid, and point-of-care solutions.^2^^,^^3^^,^^4^ However, due to the mismatch tolerance exhibited by Cas proteins, achieving single-nucleotide fidelity in CRISPR-based diagnostics (CRISPRdx) is not inherently straightforward.^1^^,^^5^ Overcoming this challenge requires innovative approaches to enhance specificity, which have been the focus of numerous recent studies. This protocol outlines the steps to develop a CRISPR-based test for accurate detection of single-nucleotide variants (SNVs). A specific application that we validated involves high-fidelity detection of cancer-associated SNVs, trained on synthetic DNA, cell line-derived cell-free DNA (cfDNA), and evaluated on patient-derived plasma samples. However, this protocol can also be applied to multiple clinically relevant DNA sequences of interest, found in different types of samples, including other types of (liquid) biopsies (urine, tissue biopsy, etc.) or other cell lines than those described. This protocol focuses on optimizing Cas12a-based SNV detection by fluorescence, using PCR-based or isothermal amplification of DNA targets.

### Institutional permissions

Institutional permission is required for obtaining and studying clinical samples, along with informed consent from each patient or healthy donor. For the results described in this protocol, samples were collected through biobanking, or by using anonymized left-overs from routine care, in compliance with the Declaration of Helsinki and Good Clinical Practice guidelines.

### Using ARTEMIS for gRNA design

**Timing: 1–3 h**

This protocol uses the ARTEMIS (tARgeting paThogenic Mutations In the Seed region) algorithm to design CRISPR RNAs (crRNAs) for Cas12a CRISPRdx. ARTEMIS is a bioinformatics tool that identifies single-nucleotide variants (SNVs) that can be precisely targeted by Cas12a. It scans genome-wide SNV datasets, such as ClinVar, to predict which variants are compatible with Cas12a′s PAM and seed region constraints, allowing users to design optimized crRNAs with synthetic mismatches for improved specificity. To replicate the gRNA design for this particular experiment, you must download the associated code and complementary publicly available datasets.

***Note:*** We have compiled an overview of all 6,243 genome-wide ARTEMIS hits, using human genome reference GRCh38.p14 and SNV data from the ClinVar database (downloaded on the 31^st^ of July 2021). This file is accessible online (Zenodo: https://doi.org/10.5281/zenodo.14066692) in variant call format (*.vcf*), and can be used to check whether the user’s SNV of interest is targetable with Cas12a. A subset of this list with 928 cancer-associated variants is also available from Data S1 from Kohabir et al. (2024).^1^ If the variant of interest is present, the user can skip step 1, and can continue with primer design. If it is not present, but was present in ClinVar before July 2021, it is most likely not targetable with Cas12a.

In case the variant of interest was added to ClinVar after July 2021, it is necessary to rerun the ARTEMIS pipeline. Likewise, if the user is interested in a different set of variants, PAM sequence, seed region size and/or reference genome, the ARTEMIS pipeline also has to be rerun. Step 1 illustrates how to apply the ARTEMIS pipeline on the ClinVar database.

1. Install the ARTEMIS pipeline as a command line utility in a Linux environment.
  a. Download and unzip ARTEMIS package: https://doi.org/10.5281/zenodo.14627118.
  b. Download databases: ClinVar (available at https://www.ncbi.nlm.nih.gov/clinvar/), GRCh38 (through ensemble https://ftp.ensembl.org/pub/release-113/fasta/homo_sapiens/dna/ or NCBI https://www.ncbi.nlm.nih.gov/datasets/genome/GCF_000001405.40/).
  c. Additionally download 1000 genomes data to exclude commonly mutated PAM sites: ftp://ftp.1000genomes.ebi.ac.uk/vol1/ftp/data_collections/1000_genomes_project/.
  d. Change directory into the top level of the unzipped archive and install using pip: “python3 -m pip install’’.

***Note:*** ARTEMIS can be run as a command line utility from any directory, as follows: “artemis <reference> <variants> > cas12targetable.vcf”, where <reference> should be a path to a FASTA file (e.g. GRCh38) and <variants> a *.vcf* file (e.g. ClinVar). The output is written to standard out and, with this example would be written to file called cas12targetable.vcf. For more information we refer to the readme file in the package and the help that is included with the command line utility.

**CRITICAL:** Genome builds and contig names should match between the databases. Executing the code will result in a list of genome-wide hits as formatted by the input .*vcf* (e.g. ClinVar), but with additional INFO fields for the relative location of the PAM site, reference and altered allele gRNA target sequence.

***Optional:*** For this protocol, the Cas12a seed region is defined as the pentanucleotide stretch flanking the 3′ side of the PAM. The Cas12a PAM sequence is defined as TTTV. The user can alter the PAM preferences or seed region properties on the command line by specifying the optional arguments for ‘seedsize’ and ‘pamregex’.

***Optional:*** The obtained list is ready to be used for gRNA design for SNVs of interest. Hits can be further filtered for relevant keywords that occur in the ClinVar information fields, e.g. “cancer”, “melanoma” and “carcinoma”.

### Cell culture

**Timing: ∼1 week**

**Figure 1:**
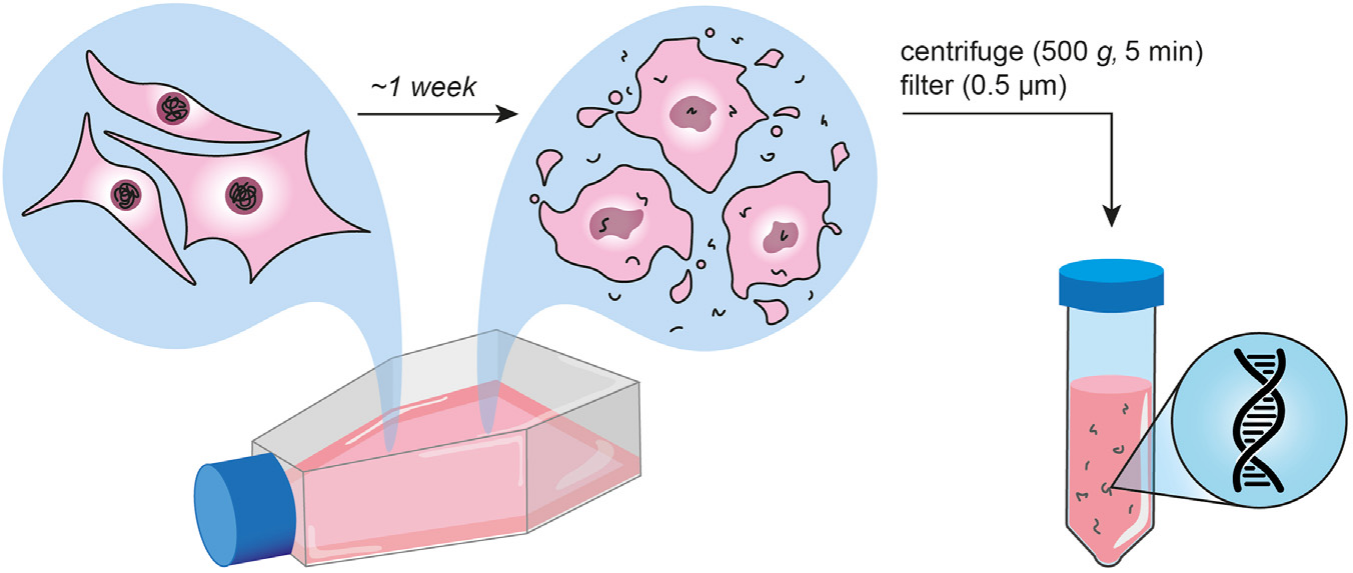
Harvesting conditioned medium for cfDNA isolation During apoptosis, genomic DNA is fragmented and subsequently released into the surrounding medium. After approximately one week of incubation, the conditioned medium can be collected by centrifugation and filtration, ensuring the removal of any viable cells with intact genomic DNA from the final harvested medium.

This protocol assesses crRNA specificity on different targets, including synthetic oligonucleotides and cfDNA derived from conditioned cell culture medium (Figure 1). Growing relevant cell lines and harvesting medium in advance can save time when crRNAs show improved specificity on synthetic targets and are ready to be evaluated using cell line-derived cfDNA.

2. Select relevant cell lines and bring them into culture.
  a. Seed ∼3 million cells in corresponding medium in a T175 flask or a 15 cm cell culture dish.
  b. Place the flask/dish in a stationary incubator (37°C, 5% CO_2_).
  c. Grow the cells for ∼1 week without changing the medium.

***Note:*** Various databases (such as Cellosaurus^6^) contain information on occurrence of point mutations in characterized cell lines. This can be used to select mutant cell lines and train the SNV detection on cfDNA thereof. Alternatively, cell lines may be derived through immortalizing cells from patient biopsies in which the mutation was found or suspected.

***Note:*** This protocol used melanoma cell lines. The indicated seeding density, culture media and incubation times can vary for other cell types for sufficient cfDNA secretion into the conditioned medium, see troubleshooting, problem 2

***Note:*** Cell lines may have complex karyotypes that can cause misinterpretation of results (See troubleshooting, problem 4).

3. Harvest the conditioned medium.
  a. Collect the conditioned medium in a falcon tube, suitable for centrifugation.
  b. Centrifuge the conditioned medium at 500 *g* for 5 min.
  c. Transfer the supernatant into a new tube, discard the debris.
    ***Optional:*** Filtering the supernatant through a 0.45 μm sterile filter can further reduce the amount of cellular debris and macromolecular DNA, yielding a better mimic of fragmented cfDNA.
  d. Store the processed conditioned medium at −20°C until further use.

### Obtaining and preparing liquid biopsies

**Timing: 30 min**

This protocol makes use of blood-based liquid biopsies to evaluate the performance of an *in vitro* CRISPR-based test. In order to compare the sensitivity and specificity of the test, it is recommended to subject the clinical isolates to standard diagnostic techniques (e.g., qPCR, sequencing).

**Figure 2:**
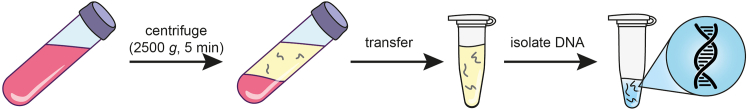
Isolation of cfDNA from blood Non-coagulated blood is centrifuged to harvest the resulting plasma supernatant, containing cfDNA. cfDNA can be isolated from plasma using affinity-based purification protocols, such as QIAsymphony DSP DNA mini as described in this protocol.

4. Prepare plasma samples from collected blood in tubes containing an anti-coagulant (e.g., EDTA) (Figure 2).
  a. Centrifuge K2EDTA-plasma at 2500 *g* for 5 min at 20°C–25°C .
  b. Transfer the plasma supernatant to a new tube.
  c. Store at −20°C until use.

***Optional:*** This protocol describes steps for cfDNA isolation from plasma. Alternatively, cfDNA can be isolated from serum (supernatant after blood coagulation). This material is known to contain significantly more genomic DNA from white blood cells, which can result in a lower fraction of the variant of interest in the cfDNA isolate. Unless the user is interested in detecting germline variants, this may lower the signal-to-noise ratio due to increased wild type background material.

5. Isolate the cfDNA using QIAsymphony SP instrumentation, with the QIAsymphony DSP DNA mini kit, according to the manufacturer’s instructions (Qiagen).
  a. Perform extraction on 3.2 mL aliquots.
  b. Elute in ∼50 μL volume per extraction.
  c. Pool all DNA isolates that originate from the same liquid biopsy sample.

***Note:*** This protocol describes steps for automated DNA extraction using QIAsymphony SP. Alternative DNA isolation methods can be used as well, including column-based methods, taking into account that the technique purifies nucleosomal cfDNA fragment sizes (∼160 nt) efficiently.

6. Proceed to quantify the double-stranded DNA (dsDNA) content of each pooled isolate, using Qubit High Sensitivity dsDNA kit, according to the manufacturer’s instructions.

## Key resources table

**Table:** 

REAGENT or RESOURCE	SOURCE	IDENTIFIER
**Chemicals, peptides, and recombinant proteins**		

Roswell Park Memorial Institute (RPMI) medium	Gibco	Cat#11875093
Iscove’s modified Dulbecco’s medium (IMDM)	Gibco	Cat#12440053
Dulbecco’s modified Eagle’s medium (DMEM)	Gibco	Cat#10566016
Fetal bovine serum (FBS)	HyClone	Cat#SH30071.03
SuRE/Cut buffer M	Roche	Cat#11417983001
rCutSmart buffer	New England Biolabs	Cat#B6004S
EnGen LbCas12a	New England Biolabs	Cat#M0653T
dNTP	New England Biolabs	Cat#N0447L

**Critical commercial assays**		

QIAsymphony	QIAGEN	Cat#9001301; Cat#937236
Qubit dsDNA HS kit	Invitrogen	Cat#Q32851
Q5 Hot Start High-Fidelity DNA Polymerase	New England Biolabs	Cat#M0493L
TwistAmp Liquid Basic RPA kit	TwistDx	Cat#TALQBAS01
QIAquick PCR Purification Kit	QIAGEN	Cat#28104
Mix2Seq	Eurofins	N/A

**Deposited data**		

ClinVar database	Landrum et al.^7^	N/A
1000 Genomes variant call-set	Zheng-Bradley et al.^8^	N/A

**Experimental models: Cell lines**		

Human cell line SKmel28	ATCC	Cat#HTB-72; RRID: CVCL_0526
Human cell line WM9	Rockland	Cat#WM9-01-0001; RRID: CVCL_6806
Human cell line VU1131	Prof. R. Brakenhoff (Amsterdam UMC)	Cat#VU-SCC-1131; RRID: CVCL_XX18
Human cell line Mel25	Prof. T.D. de Gruijl (Amsterdam UMC)	N/A
Human cell line Mel28	Prof. T.D. de Gruijl (Amsterdam UMC)	N/A
Human cell line Mel78	Prof. T.D. de Gruijl (Amsterdam UMC)	N/A
Human cell line Mel79	Prof. T.D. de Gruijl (Amsterdam UMC)	N/A
Human cell line Mel84	Prof. T.D. de Gruijl (Amsterdam UMC)	N/A
Human cell line Mel88	Prof. T.D. de Gruijl (Amsterdam UMC)	N/A

**Oligonucleotides**		

Primers for PCR/RPA amplification	IDT	Designed by user
Cas12a crRNAs for CRISPRdx	IDT	Designed by user
Fluorescent ssDNA reporter for CRISPRdx:/56-FAM/TTATT/3IABkFQ/	IDT; Chen et al.^2^	N/A

**Software and algorithms**		

ARTEMIS code	Kohabir et al.^1^	Zenodo: https://doi.org/10.5281/zenodo.14627118
Prism	GraphPad Software LLC	v.10.2.0

**Other**		

Infinite plate reader	Tecan Group Ltd.	Infinite 200 Pro M Plex
i-control	Tecan Group Ltd.	v.2.0
Low volume 384-well black flat bottom polystyrene NBS microplate	Corning	Cat#3820
DNase1	New England Biolabs	Cat#M0303

## Materials and equipment

• This protocol uses the Infinite 200 Pro M Plex platereader and complementary i-control software (Tecan Group Ltd.) but should be adapted according to guidelines when using comparable equipment from other manufacturers. To follow this protocol, the platereader should be able to (i) quantify green fluorescence over time, (ii) incubate at 37°C, and (iii) ideally enable the user to indicate a selection of wells to be monitored.
  ○ Exact instructions on how measurement protocols can be programmed can be found in the manufacturer’s instructions. This protocol makes use of single well-directed kinetic cycles, with a kinetic interval of 5 min. Using these settings, the run monitors fluorescence in each indicated well, every 5 min.
  ○ This protocol uses Low Volume 384-well Black Flat Bottom Polystyrene NBS (Corning Inc.) microplates to monitor 20 μL final volume reactions
  ○ The following settings need to be used, as calibrated for the assay concentrations described in this protocol.
    - Mode: Fluorescence top reading
    - Temperature: 37°C
    - Excitation: 485 nm
    - Emission: 535 nm
    - Excitation bandwidth: 9 nm
    - Emission bandwidth: 20 nm
    - Gain: 85 (manual)
    - Number of flashes: 10
    - Integration time: 20 μs
    - Lag time: 0 μs
    - Settle time: 0 μs
    - Z-position (manual): 16814 μm
  ○ Kinetic data is written most conveniently by representing data from different wells in columns, and time points as rows. This can be installed before starting the run through Settings > Results Presentation > Kinetic > Rotation: column-wise.
  ○ For more convenient fluorescence data processing, run replicate reactions adjacent and unidirectional in the microplate. The i-control software can write the data of these replicate reactions in adjacent columns in the Excel spreadsheet, which allows for more convenient data processing. This can be installed before starting the run through Settings > Results Presentation > Kinetic > Align: A1B1 (arranged by the columns in the microplate) or A1A2 (arranged by the rows in the microplate).
  ○ As a positive control for testing the technical setup, the user may validate the complete degradation of 100 nM FAM-fluorescent reporter by adding 2 U DNase1 (New England Biolabs) in buffered conditions and monitor fluorescence signal over time. Note that DNase1 requires Ca^2+^, which is not present in the CutSmart buffers used in this protocol. Instead, use the supplied buffers that come with the enzyme, or other restriction buffers that contain Ca^2+^.

## Step-by-step method details

### crRNA and primer design

**Timing: 1 h**

This part of the protocol describes the design of synthetic mismatch-engineered crRNAs for specific detection of SNV hits found by ARTEMIS. Additionally, this part gives instructions for the design of synthetic DNA targets to later evaluate the specificity *in vitro.* This protocol will focus on *BRAF* p.V600E detection as example; the user can apply the same strategies to other SNV targets of interest.

1. Design the crRNAs with and without synthetic mismatches.
  a. Identify *in silico* which PAM site corresponds to the SNV tracked by ARTEMIS.
    ***Note:*** The PAM site must be within five nucleotides distance upstream of the SNV, as defined by the Cas12a seed region (Figure 3A). This protocol uses a TTTV PAM site.

**Figure 3:**
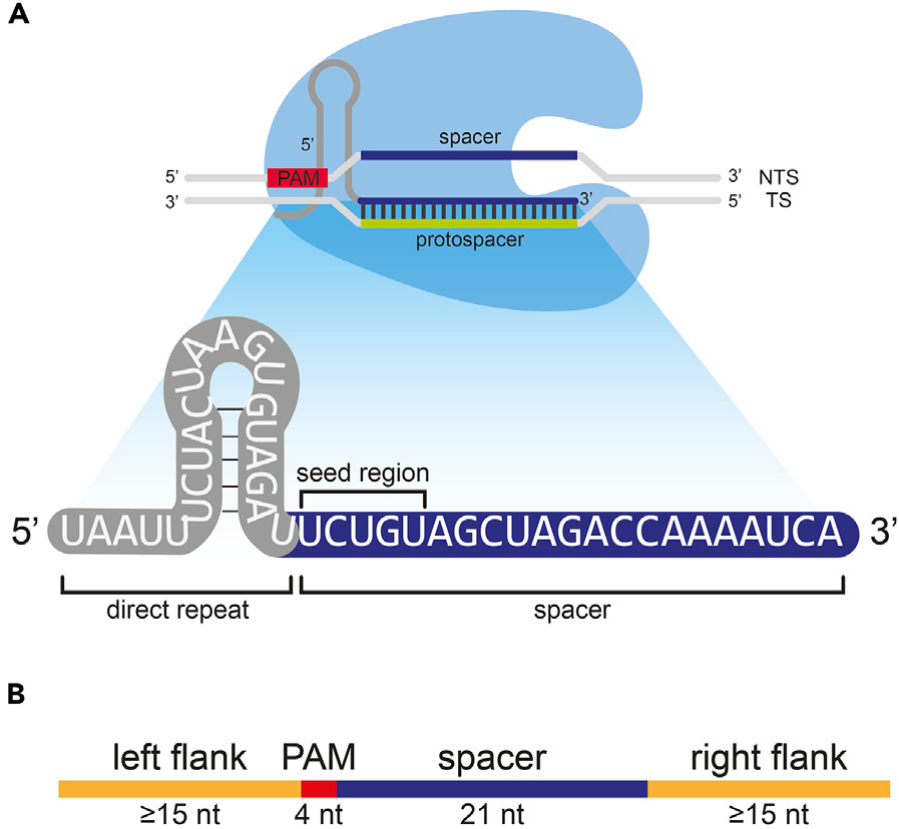
Overview of crRNA design for LbCas12a-based detection (A) Cas12a interrogates dsDNA using the spacer sequence of the crRNA that hybridizes with the protospacer sequence on the DNA target strand [TS]. The first 5′ pentanucleotide stretch of the crRNA spacer forms the seed region. The shown direct repeat sequence is compatible with LbCas12a. The displayed spacer sequence corresponds to BRAF p.V600E, without synthetic mismatches. (B) Schematic overview of designed synthetic DNA oligonucleotide targets that contain ends with flanking genomic sequences.
  b. Identify the corresponding 21 nt spacer sequence, 3′ flanking the PAM site.
    ***Optional:*** This protocol uses 21 nt spacers. Cas12a uses crRNAs with spacers that target ∼20 nt downstream of the PAM site.
  c. Design a single synthetic mismatch in the seed region to improve the crRNA specificity.
    **CRITICAL:** Do not introduce a synthetic mismatch at the position where the SNV must be recognized. Synthetic mismatches can be placed anywhere else in the seed region to improve specificity.^1^ To find the optimal positioning, multiple positions can be tested and evaluated.
    ***Note:*** For a given position, different introduced mismatched nucleobases can have varying efficiency. We have used synthetic mismatched resulting in homopairs (e.g. G·G, A·A, U·T, C·C), which resulted in improved specificity. See troubleshooting, problem 3.
  d. Order the designed crRNAs.
    ***Note:*** Make sure that the crRNA scaffold is compatible with the used Cas12 effector. This protocol is designed for LbCas12a-based detection, which uses a 5′ UAAUUUCUACUAAGUGUAGAU scaffold sequence.
    ***Optional:*** crRNAs can be ordered with protective terminal chemical modifications against ribonucleases. This protocol used Alt-R™ proprietary chemically modified crRNAs (on both ends) from Integrated DNA Technologies, Inc.
2. Design the synthetic target oligonucleotides to test crRNA specificity.
  a. Localize PAM and target sequence of interest on the reference genome and select both, with at least 15 nucleotides flanking upstream of the PAM and downstream of the target site.
  b. Design the reverse complement of the [≥15 nt]-[PAM]-[target sequence]-[≥15 nt] sequence defined under step 2a (Figure 3B).
  c. Order both sequences defined under steps 2a and 2b as single-stranded DNA (ssDNA) oligonucleotides.

**CRITICAL:** For each relevant SNV, design two different synthetic testing targets (mutant and wild type). In other words, for each SNV of interest, the user orders two complementary ssDNA pairs, a total of four ssDNA oligonucleotides. Do not design the positions of synthetic mismatches within the synthetic DNA targets, only in the crRNA spacer seed region.

***Optional:*** The artificial DNA targets can also be ordered as dsDNA oligonucleotides, in that case step 2b can be omitted, and the user should order the [≥15 nt]-[PAM]-[target sequence]-[≥15 nt] sequence defined under step 2a as dsDNA.

### Testing on synthetic oligonucleotides

**Timing: <6 h**

**Figure 4:**
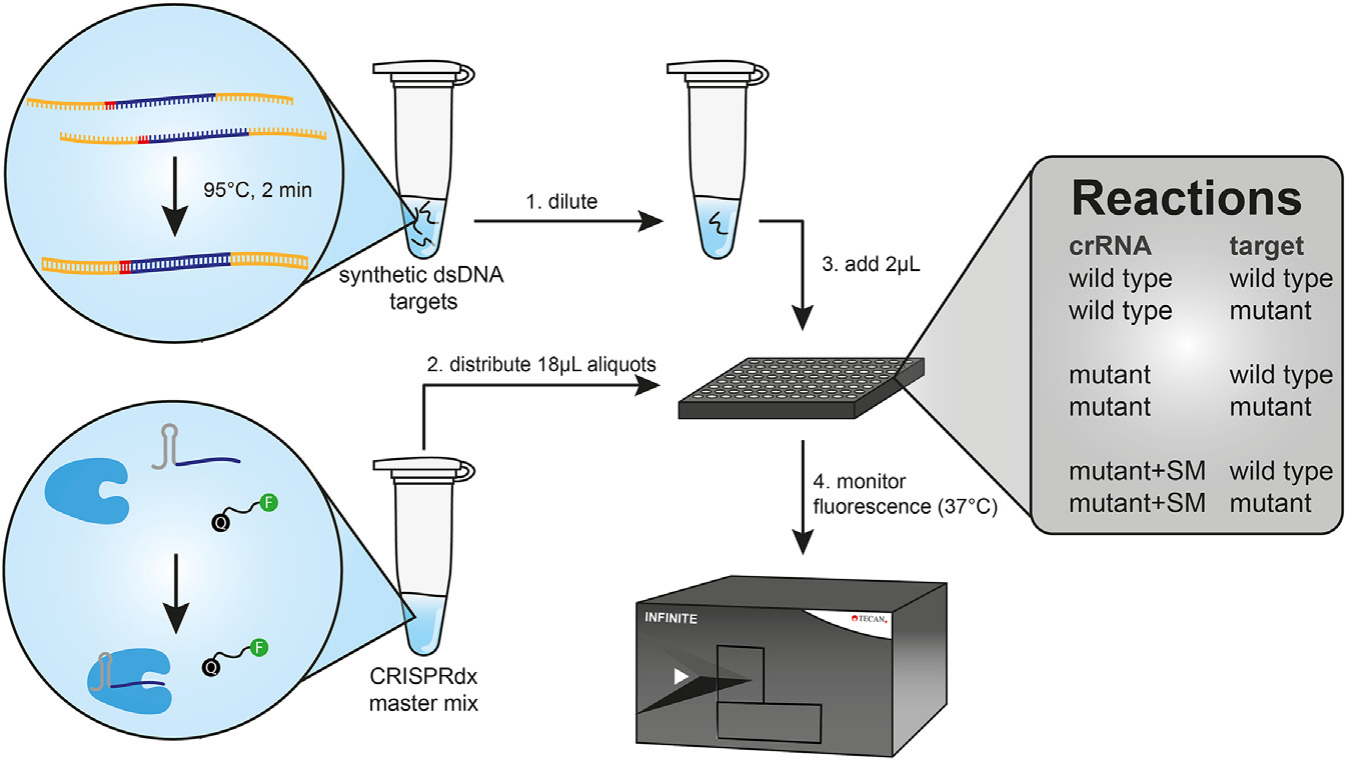
Schematic overview of CRISPRdx testing on synthetic dsDNA oligonucleotides Duplex DNA formation of the ordered synthetic ssDNA nucleotides yields dsDNA targets that can be diluted to desired concentrations. CRISPRdx mastermix, consisting of Cas12a, crRNA and reporter in a buffered solution is distributed in a 384-well plate, followed by addition of target. Different combinations of crRNA and target will have to be tested to evaluate specificity improvement after engineering a crRNA with a synthetic mismatch [SM]. Reactions are monitored for fluorescence over time in a pre-heated plate reader.

This part of the protocol describes how synthetic oligonucleotides can be used to assess whether crRNAs confer single-nucleotide specificity to the CRISPRdx reaction (Figure 4).

3. Formation of synthetic dsDNA targets.
  a. Mix complementary ssDNA oligonucleotides in equimolar ratio in a buffered solution (Table 1).
    ***Note:*** This protocol uses 1x SuRE/Cut buffer M (Roche), but this can be replaced by other buffers that stabilize DNA under slightly basic conditions (e.g. Tris-Cl, pH 8.0 or TE buffer).

**Table 1:** Composition of reaction mixture for the formation of 10 μM dsDNA targets in 50 μL

Reagent	Stock concentration	Amount
ssDNA oligo 1	100 μM	5 μL
ssDNA oligo 2	100 μM	5 μL
SuRE/Cut buffer M	10x	5 μL
Water	-	35 μL
  b. Heat the mixture to 95°C for 2 min.
  c. Cool down 0.1 °C/s to ambient temperature to form duplex DNA.
  d. Dilute the obtained mixture to desired concentrations in 1x buffer.
    ***Note:*** Cas12a had an amplification-free detection limit in the picomolar range. We recommend using target dilutes between the picomolar and attomolar levels when performing sensitivity testing with target amplification through e.g. PCR or RPA.
  e. Store at −20°C until further use.
    ***Note:*** Low DNA concentrations and poorly buffered solutions can result in increased oligonucleotide degradation, even at −20°C, see troubleshooting, problem 1.
4. Preparation of CRISPRdx mastermix.
  a. Mix the different components for the CRISPRdx mastermix (Table 2).
    ***Note:*** Take into consideration at least three replicates per reaction, and prepare a ∼10% surplus of mastermix. If needed, use diluted stock concentrations that can be pipetted more accurately.
    **CRITICAL:** Do not pipette synthetic target DNA into the reaction mix, as this will trigger Cas12a to already cleave the reporter in the mastermix.
    ***Note:*** This protocol uses CutSmart buffer, which can be replaced with other restriction buffers. Alternative buffers must at least contain 1 mM of Mg^2+^ in order for Cas12a to function. Do not use buffers with chelating agents such as EDTA, as this quenches the reaction.
    ***Note:*** Include at least three replicates for a negative control reaction, in which water is added instead of target DNA.
    ***Optional:*** Incubate the mastermix ∼15 min on bench to allow ribonucleoprotein formation. However, to save time and reduce protocol steps, this can be skipped since ribonucleoprotein formation also sufficiently takes place in the CRISPRdx reaction. Occasionally, this may lead to slightly longer lag phase of fluorescence signal growth.

**Table 2:** Composition of the mastermix reaction mixture for CRISPRdx with 40 nM Cas12a

Reagent	Stock concentration	Final concentration	Amount per reaction
CutSmart Buffer	10x	1x	2 μL
Water	-	-	15.62 μL
FAM reporter	10 μM	100 nM	0.2 μL
crRNA	10 μM	50 nM	0.1 μL
EnGen LbCas12a	10 μM	40 nM	0.08 μL
5. Install the setup for the fluorescence detection.
  a. Turn on the plate reader and make sure the settings are adjusted for monitoring FAM-based fluorescence over time. See materials and equipment setup.
  b. Prepare for the next step by switching on the heating of the plate reader to 37°C.
  c. Plan the setup of the 384-well plate.
    ***Note:*** This is critical for larger amounts of parallel reactions. We recommend to use a multichannel pipette for distributing the master mix and targets in step 6. Therefore, distribute the master mix and targets accordingly over a strip of PCR tubes, and take into consideration the setup of the wells.
    ***Note:*** A 12 or 8 multichannel pipette may skip wells when used for a 384-well plate.
    ***Optional:*** If using i-control software (Tecan Group Ltd.), select the wells that will be used.
    i. Vortex and spin down the PCR tube strips briefly. Keep the lids on until further use.
  d. Make sure that the scanning mode is set for a kinetic measurement and indicate the total duration and scanning interval timing.
    ***Note:*** We suggest using a 5-min interval for at least 3 h.
6. Run the CRISPRdx reaction.
  a. Vortex the master mix shortly, spin it down and distribute 18 μL aliquots in a 384-well plate, in the wells selected under step 5c.
    ***Note:*** This is more easily and consistently done using a multichannel pipette.
  b. Add 2 μL of each synthetic oligonucleotide dsDNA sample in destined wells. Pipette up and down 5 times to mix well.
    i. For the negative control reactions add water instead of DNA.
      **CRITICAL:** To ensure simultaneous reaction initiation, use a multichannel pipette to add target DNA to the reactions.
      ***Optional:*** Tap the plate lightly on the bench, to remove bubbles. This is optional as long as the mixture is well-mixed. With the described heated platereader setup, bubbles will resolve over time and cause minimal noise to the bottom-read fluorescence.
  c. Place the plate in the plate reader and start the measurement protocol.
7. Monitoring the reaction.
  a. As soon as the run starts, i-control software automatically opens and writes acquired data in an excel file spreadsheet.
  b. If the fluorescence values do not increase any further, the run can be aborted and the data can be saved. Otherwise, let the run finish.
  c. Save the spreadsheet for further analysis, see quantification and statistical analysis.

### Testing on cfDNA derived from cell lines

**Timing: ∼1 day**

When synthetic mismatches result in increased specificity on synthetic targets, the test can be further developed for detection in samples with a genomic background. Additionally, this is where amplification can be integrated into the workflow.

8. Design primers for PCR amplification.
  a. With the target site(s) defined in step 1b, design a pair of primers for PCR amplification of the locus. Primer design can be done with primer design software such as SnapGene and Benchling.
    ***Optional:*** Alternatively, the user may design primers for isothermal amplification through e.g. RPA using the TwistAmp Liquid Basic RPA kit (TwistDx). In that case, follow the primer design considerations as instructed by the manufacturer.
    ***Note:*** Depending on the final application, the target of interest may occur on highly fragmented DNA, which is the case for cfDNA found in most liquid biopsies. When designing primers, aim for short amplicons of 60–120 bp to increase the chances of occurring on a single DNA fragment. Primer sequences may not overlap with the target sequence, to prevent Cas12a from getting falsely activated by the primers.
  b. Confirm that the designed primer pair does not form hetero/homo-dimers and has suitable melting temperatures and GC-content for the aimed amplification method. Primer sequences can be screened for undesired dimerization using webtools such as Thermo Fisher – Multiple Primer Analyzer or IDT- OligoAnalyzer.
  c. Order the primers.
9. Isolate cfDNA from conditioned cell culture medium.
  a. Thaw the prepared conditioned medium (see before you begin - cell culture)
  b. Proceed to isolate the cfDNA using QIAsymphony SP & QIAsymphony DSP DNA Mini kit, according to the manufacturer’s instructions (Qiagen).
    i. Perform extraction on 3.2 mL aliquots.
    ii. Elute in ∼50 μL volume.
    iii. Pool all DNA isolates that originate form the same conditioned medium.

***Note:*** This protocol describes steps for automated DNA extraction using QIAsymphony SP. Alternative DNA isolation methods can be used as well, including column-based methods, taking into account that the technique purifies nucleosomal fragment sizes (∼160 nt) efficiently.

10. Quantify the pooled cfDNA isolates as prepared under step 9.
  a. Proceed to quantify the dsDNA content, using Qubit High Sensitivity dsDNA kit, according to the manufacturer’s instructions.

**Pause Point:** Pooled isolates can be stored at −20°C until further use. For further use, thaw the isolates at ambient temperature.

11. Perform nucleic acid amplification.
  a. In case the user has access to thermocycling machinery, prepare and run 15 μL PCR reactions (Tables 3 and 4). Alternatively, choose step 11b for isothermal amplification
    ***Optional:*** In case of multiple PCR reactions, prepare a master mix without adding DNA template, distribute 13 μL aliquots in PCR tubes and add 2 μL template individually.
    i. As template, use cfDNA isolates from conditioned cell medium as prepared & quantified under steps 9 and 10. Include a PCR reaction with nuclease-free water as template, as a negative control.

**Table 3:** Composition of a 15 μL PCR reaction mixture

Reagent	Stock concentration	Final concentration	Amount per reaction
DNA template	variable	variable	2 μL (0.5–10 ng)
Q5 DNA Polymerase	2000 U/mL	20 U/mL	0.15 μL
forward primer	10 μM	500 nM	0.75 μL
reverse primer	10 μM	500 nM	0.75 μL
dNTP	10 mM	200 μM	0.3 μL
Buffer	5x	1x	3 μL
Nuclease-free water	-	-	8.05 μL

**Table 4:** Overview of thermocycling conditions used for amplification with primer pair KD217 and KD219

Steps	Temperature	Time	Cycles
Initial Denaturation	98°C	30 s	1
Denaturation	98°C	10 s	28 cycles
Annealing	60°C	20 s	
Extension	72°C	15 s	
Final extension	72°C	2 min	1
Hold	4°C	forever
  b. In case the user prefers isothermal amplification, prepare and run 15 μL RPA reactions (Tables 5 and 6).
    ***Optional:*** In case of multiple RPA reactions, prepare a mastermix without adding DNA template and MgOAc. Distribute 12.25 μL aliquots in PCR tubes and add 2 μL template individually.
    i. As template, use cfDNA isolates from conditioned cell medium as prepared & quantified under steps 9 and 10. Include a RPA reaction with nuclease-free water as template, as a negative control.
      **CRITICAL:** RPA reactions start as soon as MgOAc is added to the reaction. Therefore, add the MgOAc in the PCR tube lid and close carefully. Spin down the PCR tube strip to ensure simultaneous start of amplification across the different reactions.
      ***Optional:*** In case the SNV status of the used cell line is unknown, the user may want to verify the zygosity through Sanger sequencing the PCR or RPA product, using the QIAquick PCR Purification Kit (Qiagen) and Mix2Seq kit (Eurofins). In that case, take into account a larger PCR or RPA reaction volume.

**Table 5:** Composition of RPA reaction mixtures

Reagent	Stock concentration	Final concentration	Amount per reaction
DNA template	variable	variable	2 μL (0.5–10 ng)
Reaction buffer	2x	1x	7.5 μL
Forward primer	100 μM	467 nM	0.07 μL
Reverse primer	100 μM	467 nM	0.07 μL
dNTP	10 mM	900 μM	1.35 μL
E-mix	10x	1x	1.5 μL
Nuclease-free water	-	-	1 μL
Core mix	20x	1x	0.75 μL
MgOAc	280 mM	14 mM	0.75 μL

**Table 6:** Overview of incubation conditions used for RPA with primer pair KD117 and KD118

Steps	Temperature	Time	Cycles
Isothermal amplification	37°C	20 min	1
Heat inactivation	80°C	2 min	1
Hold	4°C	forever
12. Prepare the CRISPRdx reaction as described in step 4.
13. Install the setup for the fluorescence detection as described in step 5.
14. Run the CRISPRdx reaction as described in step 6. Instead of synthetic oligonucleotide dsDNA, add 2 μL PCR or RPA reaction mixture as activator.

**CRITICAL:** To ensure simultaneous reaction initiation, use a multichannel pipette.

***Optional:*** Tap the plate lightly on the bench, to remove bubbles.

15. Monitor the CRISPRdx reaction as described in step 7.

### Testing on liquid biopsy material

**Timing: ∼4 h**

Detection with single-nucleotide fidelity on amplified cfDNA from conditioned cell culture medium does not entirely reflect actual liquid biopsy-derived cfDNA, in which the tumor fraction is often very low. Testing on actual liquid biopsy samples will gain more insight into the performance of the test in its destined setting.

16. Thaw liquid biopsy plasma samples as prepared (see before you begin - obtaining and preparing liquid biopsies).
17. Perform nucleic acid amplification as described in step 11.
  a. Instead of adding 2 μL cell line derived cfDNA, add 2 μL plasma directly.
18. Prepare the CRISPRdx reaction as described in step 4.
19. Install the setup for the fluorescence detection as described in step 5.
20. Run the CRISPRdx reaction as described in step 6.
  a. Instead of synthetic oligonucleotide dsDNA, add 2 μL PCR or RPA reaction mixture as activator.

**CRITICAL:** To ensure simultaneous reaction initiation, use a multichannel pipette.

***Optional:*** Tap the plate lightly on the bench, to remove bubbles.

21. Monitor the CRISPRdx reaction as described in step 7.

## Expected outcomes

Expected cell line cfDNA yields after QIAsymphony isolation can vary significantly between cell lines and culture volume. Our study used melanoma cell lines, generated from metastatic melanoma biopsies. We isolated roughly ∼5–12 ng cfDNA per mL conditioned medium. If using other cell lines, cfDNA yields may be different.^9^

The expected fluorescence yield is largely dependent on the amount and condition of the fluorescent reporter used. With the particular setup as described in this protocol (100 nM reporter in 20 μL reaction volume, with the indicated settings on an Infinite 200 Pro M Plex platereader [Tecan Group Ltd.]), maximum fluorescence signal ranges between 4000-5000 arbitrary units (A.U.).

## Quantification and statistical analysis

**Timing: 1 h**

The saved Excel file contains all raw fluorescence data from the kinetic measurement, listed together with other settings (time, software version, plate type, run protocol, predefined measurement settings and which wells of the plate were measured. For each well, the raw fluorescence data is organized column-wise in the Excel spreadsheet. Depending on the i-control software settings (see materials and equipment setup), the data from replicate reactions is written in adjacent columns. Adjacent columns representing replicates can be copied and pasted into alternative analysis software.

**Figure 5:**
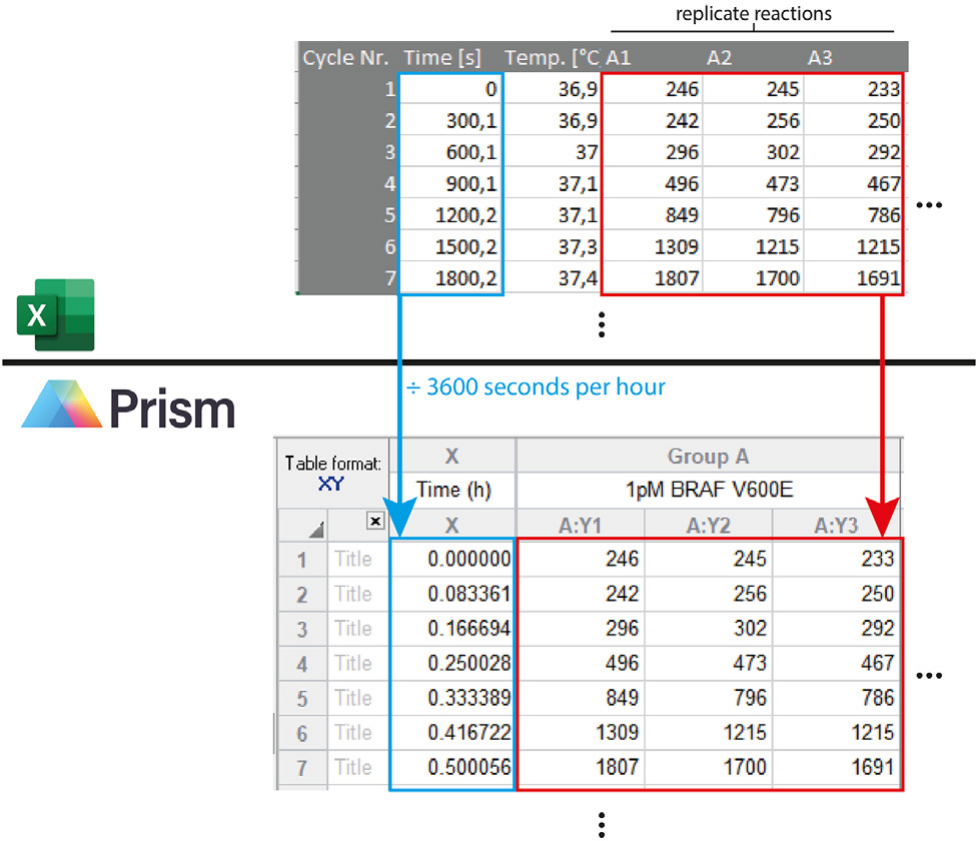
Transfer of raw data from excel to GraphPad Prism software Raw fluorescence data is pasted into a XY table format, with replicates represented as adjacent columns. Time units have been adjusted accordingly.

This protocol uses GraphPad Prism software for visualization and statistical analysis (Figure 5). GraphPad Prism allows for statistical analyses based on *n* replicates, placed in side-by-side columns, where *n* is defined by the user when opening a new project file. As the kinetic measurement displays time-dependent data, *XY* format is the most suitable for complete visualization of the data. Mean values are then plotted against time, automatically displaying error bars. Fluorescence ratios are calculated by clicking *New Analysis* under the Results section in the left-hand panel, and selecting “Remove baseline and column math” in the window that appears. Data can be normalized to one set of replicates or to every other set of replicates (even or uneven). Calculated ratios and standard deviations can be copied and pasted into a new data table, and plotted as bar charts.

When comparing endpoint fluorescence values only, data may be best visualized using a *Column* or *Grouped* format. In that case, significant differences in fluorescence can be calculated using two-way ANOVA. The endpoint data should be organized in such a way that rows contain different crRNAs and columns contain different tested samples/targets/concentrations. Two-way ANOVA is done by clicking *New Analysis* under the Results section in the left-hand panel, and selecting “two-way ANOVA (or mixed model)” in the window that appears. Pair-wise comparisons can automatically be added in the graph, displaying the *p-*values.

## Limitations

A major limitation of the use of Cas12a is the dependency on a PAM sequence being in close proximity to SNVs of interest. Although some Cas12a proteins have been engineered for a broader TTTN PAM preference, these also show to be less specific when it comes to distinguishing single nucleotide differences.^5^ Another limitation of this protocol is the lack of control on unforeseen genetic variants that can lead to mismatches at a different position elsewhere in the crRNA:DNA heteroduplex, thereby influencing the reaction kinetics. This has been shown to reduce the efficiency of the reaction, ultimately resulting in reduced sensitivity.^1^ If the unforeseen variant occurs within the seed region, or in the PAM sequence, this may even lead to complete incapacitation of target recognition. This protocol introduces synthetic mismatches in crRNAs that result in homopairs in the heteroduplex (e.g., A·A, G·G, C·C, U·T). It was demonstrated this works well to enhance the specificity, but exact rationale behind the optimal synthetic mismatches has not been determined, although wobble base-pairing should be avoided.

## Troubleshooting

### Problem 1

Synthetic DNA targets do not yield sufficient CRISPRdx signal (Related to step-by-step method details – Step 3).

Cas12a detects dsDNA with better affinity compared to ssDNA. The annealed synthetic constructs may not have formed duplex DNA efficiently or may have degraded over time. This is more likely as the annealed product is older, has a lower concentration, or is badly buffered.

### Potential solution

Mixtures can be re-annealed by repeating step 3 of the protocol. However, to avoid the risk of endonucleolytic degradation over time, it is recommended to replace samples with newly annealed oligonucleotides from the principle ssDNA stocks.

### Problem 2

The cfDNA yield is very low after extraction from conditioned medium (Related to before you begin – Step 2).

Different cell lines secrete cfDNA into the medium at different rates.^9^ Too little cfDNA can mean that there were not enough cells in culture to result in significant cfDNA release upon apoptosis.

### Potential solution

Culture more cells for a longer period in without refreshing the medium. This should increase the amount of dying cells, and may yield more cfDNA.

### Problem 3

The synthetic mismatch that was introduced did not result in significantly improved specificity (Related to step-by-step method details – Step 1).

Indeed, synthetic mismatches have varying effects, depending on the position and the changed base.

### Potential solution

Testing multiple positions and selecting the most effective engineered crRNA can improve chances of improved specificity. Be cautious when introducing uracil bases in the crRNA, as these can sometimes result in semi-stable Wobble base pairs.

### Problem 4

While testing for mutant and wild type alleles on cfDNA from a heterozygous cell line, the obtained CRISPRdx fluorescence signal is not equal for both alleles (Related to before you begin – Step 3).

Cell lines can have complex karyotypes, potentially resulting in an imbalanced heterozygosity, leading to a difference in CRISPRdx signal for each allele.

### Potential solution

In principle this is not a problem, but it is important to verify this using sequencing. Determine the allele ratio. Kohabir et al. (2024) used NGS for this and showed that the ratio of the alleles corresponded to the ratio of the CRISPRdx signal.^1^

## Resource availability

### Lead contact

Further information and requests for resources and reagents should be directed to and will be fulfilled by the lead contact, Erik A. Sistermans (e.sistermans@amsterdamumc.nl).

### Technical contact

Technical questions on executing this protocol should be directed to and will be answered by the technical contact, Kavish Kohabir (k.a.v.kohabir@amsterdamumc.nl).

### Materials availability

• This study did not generate new unique reagents.

### Data and code availability

• This paper utilized existing publicly available datasets. The SNV data are accessible at the ClinVar database^7^ (https://www.ncbi.nlm.nih.gov/clinvar/). The reference human genome (GRCh38.p14) is accessible at https://www.ncbi.nlm.nih.gov/datasets/genome/GCF_000001405.40/.
• An overview of all genome-wide SNV ARTEMIS hits has been deposited at Zenodo at https://doi.org/10.5281/zenodo.14066692 and is publicly available as of the date of publication.
• All original ARTEMIS code has been deposited at Zenodo at https://doi.org/10.5281/zenodo.14066692 and is publicly available as of the date of publication.
• Any additional information required to reanalyze the data reported in this paper is available from the lead contact upon request.

## Acknowledgments

We extend our sincere gratitude to L.O. Nooi, A.E. Katajamäki, T.D. de Gruijl, D.A. Stolk, J.G.C. Stolwijk, D.A.P. Rockx, W.H. Segerink, R.E. Boyer, and D.L.S. Sie (Amsterdam UMC, Vrije Universiteit), R. Brouwer (Unilabs, Enschede), and A.M. Molina Vargas (University of Rochester) for their invaluable contributions to the original research underlying this protocol. Their efforts in brainstorming, initiating, optimizing, supplying resources, evaluating, and providing constructive feedback were instrumental in shaping this project.

## Author contributions

Conceptualization, software, methodology, validation, formal analysis, investigation, data curation, writing – original draft, writing – review and editing, visualization, and project administration, K.A.V.K.; software, data curation, and writing – review and editing, J.L.; conceptualization (equal), funding acquisition (lead), supervision (equal), and writing – review and editing (equal), E.A.S. and R.M.F.W.

## Declaration of interests

The authors declare no competing interests.
